# Supplementary material for: Insulin-like Peptides of the Western Flower Thrips Frankliniella occidentalis and Their Mediation of Immature Development
Source: Insects. 2023 Jan 3;14(1):47. doi: 10.3390/insects14010047 (PMC9864108; doi:10.3390/insects14010047)
Supplement: Supplementary file 1 [file insects-14-00047-s001.zip › insects-2126429-supplementary.pdf]

Supplementary Information

Table S1. Hot pepper varieties used in the present study, where 'R' and 'S' indicate resistant and susceptible varieties to the tomato spotted wilt virus (TSWV), respectively.

Response to TSWV	Trade names of varieties	Suppliers
R1	Asian Jumbo F1	Asia Korea Seed
R2	Color Kang	Asia Korea Seed
R3	Bul Color	Hungnong
S1	Nok Kang	Farm Hannong
S2	Han Lim Ggwari Put F1	Asia Korea Seed

Table S2. GenBank accession numbers of the genes assessed in the present study

Genes	Accession numbers	Genes	Accession numbers
Dm-ILP1	NP_648359.1	BmA10	NP_001121787.1
Dm-IILP2	NP_524012.1	BmB1	NP_001121791.1
Dm-ILP3	NP_648360.2	BmB2	NP_001121793.1
Dm-ILP4	NP_648361.1	BmB3	BAA00674.1
Dm-ILP5	NP_996037.2	BmB4	NP_001121792.2
Dm-ILP6	NM_001272256.1	BmB5	NP_001166891.1
Dm-ILP7	NP_570070.1	BmB6	NP_001121795.1
Dm-ILP8	NM_140692.3	BmB7	NP_001121789.1
Fo-ILP1	XP_026281410.	BmB8	NP_001121790.1
Fo-ILP2	XP_026276022.1	BmB9	BAA00682.1
BmA1	Q17192.1	BmB10	NP_001121788.1
BmA2	NP_001103771.1	BmB11	NP_001121606.1
BmA3	NP_001166890.1	BmB12	NP_001121794.2
BmA4	NP_001121607.2	BmC1	NP_001119736.1
BmA5	NP_001121608.2	BmC2	NP_001119735.1
BmA6	BAA00667.1	BmD1	NP_001121635.1
BmA7	NP_001121629.2	BmE1	NP_001119733.1
BmA8	NP_001121630.1	BmF1	NP_001119734.1
BmA9	BAA00672.1	BmG1	NP_001121634.1

Table S3. Primer sequences used in the present study.

Genes	Sequences	Annealing (°C)	Product (bp)
Fo-ILP1	CGACAAGAAGTACATCCACAA	50	271
	TTAGCTGTAGGGGCGTCT		
Fo-ILP2	AAGAGTACTGGACGCCG	50	155
	CACATCGCAATAGTTCATCAG		
T7-FO ILP1	TAATACGACTCACTATAGGGAGACGACAAGAAGTACATCCACAA	50	316
	TAATACGACTCACTATAGGGAGATTAGCTGTAGGGGCGTCT		
T7-FO ILP2	TAATACGACTCACTATAGGGAGAGAGGAAGAGTACTGGACGC	50	201
	TAATACGACTCACTATAGGGAGAGTTCATCAGCTCGTCTATGG		
EF1	TCAAGGAACTGCGTCGTGGAT	50 -55	129
	ACAGGGGTGTAGCCGTTAGA		

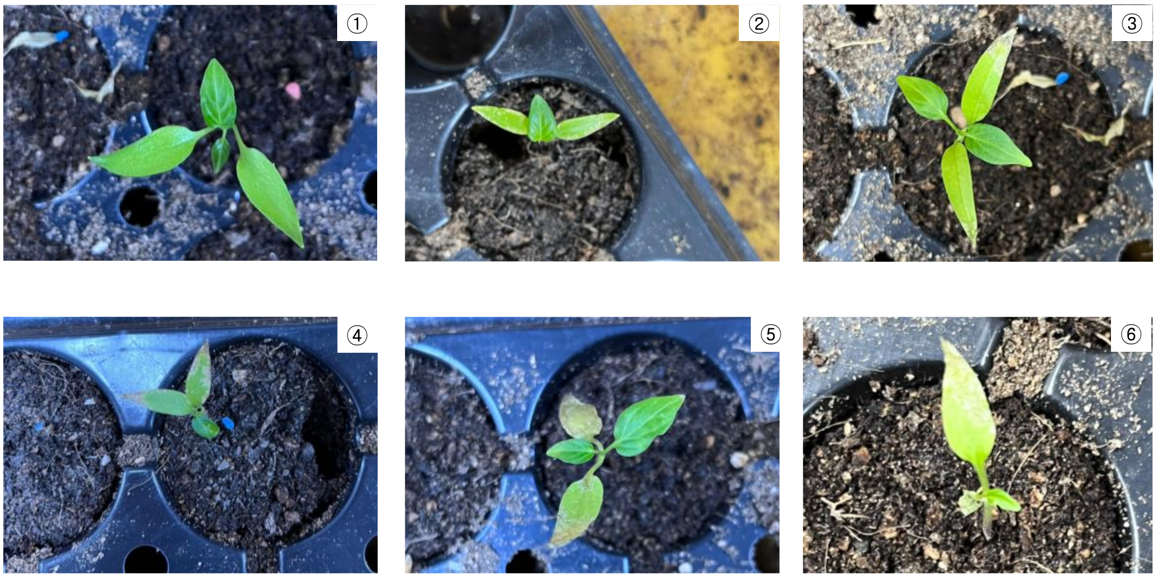

Figure S1. Evaluation of the damage intensity on a scale of 0 (no damage) to 5 (severe damage) due to thrips feeding. Damage was classified into 6 grades: no damage ① for grade 0, small damage limited to one leaf ② for grade 1, two leaves damage on the upper 1/3 ③ for grade 2, two leaves damage on the upper 1/2 ④ for grade 3, more than 2 leaves damage ⑤ for grade 4, and all leaves damaged with yellowish color ⑥ for grade 5.
